# Diabetes Management Support in Preschool and Primary School: A Qualitative Ideation Study Presenting Recommendations for Improved Communicative Practice

**DOI:** 10.3390/healthcare12020225

**Published:** 2024-01-16

**Authors:** Dan Grabowski, Lise Bro Johansen, Anne Østergaard Nannsen, Anette Andersen, Kurt Kristensen, Mia Kastrup Iken, Stine Hangaard, Mette Madsen, Anders Jørgen Schou, Sidse-Marie Toubroe, Kasper Ascanius Pilgaard, Patricia DeCosta

**Affiliations:** 1 Steno Diabetes Center Copenhagen (SDCC), Copenhagen University Hospital, 2730 Herlev, Denmark; 2 Steno Diabetes Center Aarhus (SDCA), Aarhus University Hospital, 8200 Aarhus N, Denmark; 3 Department of Pediatrics, Aarhus University Hospital, 8200 Aarhus N, Denmark; 4 Danish Diabetes Association, 2600 Glostrup, Denmark; 5 Steno Diabetes Center North Denmark (SDCN), 9000 Aalborg, Denmark; 6 Department of Pediatrics and Adolescent Medicine, Aalborg University Hospital, 9000 Aalborg, Denmark; 7 Steno Diabetes Center Odense (SDCO), 5000 Odense, Denmark; 8 Pediatric Research Unit, Odense University Hospital, 5000 Odense, Denmark; 9 Steno Diabetes Center Sjælland (SDCS), 4000 Roskilde, Denmark; 10 Department of Pediatrics, Copenhagen University Hospital, 2730 Herlev, Denmark

**Keywords:** type 1 diabetes, children, school, preschool, support, communication, recommendations

## Abstract

Diabetes care in institutional settings is a significant challenge that affects the whole family as well as care workers and teachers. The present study is the ideation part of a rigorous development process in the KIds with Diabetes in School (KIDS) project. We have previously conducted a thorough three-part needs assessment in which we explored the problem area from the viewpoints of (1) municipal administrative staff, (2) preschool and school staff and (3) families. Based on the identified needs and to a great extent on the contents and shortcomings of existing guidelines, the objective of the present study is to explore and develop possible solutions and recommendations for addressing the challenges and problems. To meet this objective, we held comprehensive multistakeholder participatory workshops in each of the five Danish regions. Five main themes with multiple subthemes were identified as areas to be addressed: (1) training and knowledge, (2) communication and collaboration, (3) the designated contact/support person, (4) national guidelines, and (5) the Diabetes Coordinator. Our findings demonstrate that communicative structures and dynamics are at the very heart of the identified problems and challenges and that the possible solutions should revolve around improving existing structures and highlighting the importance of constantly working on understanding and developing communication strategies. We propose a set of recommendations for practice based on these communicative needs.

## 1. Introduction

Type 1 diabetes (T1D) is one of the most commonly diagnosed chronic illnesses in childhood, with approximately 648,000 children (<15 years of age) living with the condition globally [1]. The incidence rate of children diagnosed is increasing, with the highest increase observed in young children [2].

The need for consistent diabetes management support is related to the cognitive development of the child, which evolves during the preschool and school years. Young children are cognitively unable to understand the cause and effect of an illness and to perform sufficient diabetes self-care without support [3,4,5,6,7].

Diabetes care in institutional settings is a significant challenge that affects the whole family as well as care workers and teachers [8,9]. Research has shown that a high level of worry is experienced by parents while their child is at the institution. The parents find it difficult to balance the uncertainty of daily life with diabetes, as they feel they need to be available during institutional hours [10,11].

As young schoolchildren have not yet developed the capacity for abstract thinking, they need help to recognize signs of hypoglycaemia, support in counting carbohydrates and administering insulin during eating situations and help to learn the fundamental skills of self-care [12,13,14]. The child requires support in self-care up until the teenage years; for some adolescents, absolute self-care can be performed, whereas others still require support [14,15]. However, noncompliance in diabetes self-care during school hours is also well-recognized in adolescence, a period characterized by omission of insulin administration and reduced testing of blood glucose, both related to the adolescent’s desire to be ‘normal’ [6,16]. Diabetes is also associated with several mental health comorbidities [17].

The present study is the ideation part of the Danish Kids with Diabetes in School (KIDS) project. We have previously conducted a three-part needs assessment in which we first explored the municipal support provided for the diabetes care of children in schools and preschools in Denmark, through a national qualitative study with 121 employees from 74 of the 98 municipalities in Denmark. This study identified four main areas of interest constituting significant deficiencies in diabetes management support: (1) institutional staff initially feel very insecure about diabetes care responsibilities, (2) there is a high degree of parental involvement and responsibilities during institutional hours, (3) the roles of health employees vary and (4) the allocation of special needs assistants (SNAs) differs significantly [18].

The second needs assessment study from the Danish KIDS project examined the organization and experiences of diabetes management in Danish primary schools as perceived by the school staff. The results indicate that most schools are capable of including children with diabetes on equal terms with their peers, as stipulated in Danish national law. The majority of schools had at least one person available to support diabetes management during the day, but the results also suggest several areas for improvement. Only a quarter of the schools have implemented specific guidelines for diabetes management, and only approximately 60% had an action plan in case of hypoglycaemia. At many of the schools, the personnel had received some diabetes-specific training. Nonetheless, they continued to experience a sense of inadequacy and a lack of readiness in various critical aspects of diabetes management [8].

The third needs assessment study found that the majority of surveyed parents, with a child with T1D, express profound concerns regarding the risk of hypoglycaemia during school hours. Furthermore, a substantial number of parents report that insufficient support impacts their work life. This study also found that only 28% of the children had a designated staff member assigned to assist in diabetes self-care during school hours. Having a designated staff member responsible for support in self-care was positively associated with parental experiences of better school–parent cooperation, better experiences of diabetes management in school and larger proportions of children and parents feeling comfortable in school. School staff support was positively associated with better parental experience of diabetes management [9].

The recently updated clinical practice consensus guidelines from the International Society for Pediatric and Adolescent Diabetes (ISPAD) [19] very competently outline how to promote optimal management of childhood and adolescent diabetes within the school environment.

In a separate chapter, ISPAD provides specific guidelines for preschool children. These do not, however, specifically address institutional preschool settings or communicative structures and roles/responsibilities [20].

In the ISPAD guidelines, it is specified that parents are responsible for informing school personnel about their child’s diagnosis, that it is the school’s responsibility to take care of the children during school hours and that parents should not be expected to compensate for inadequate school resources to attend to their child’s diabetes management during the school day [19].

The guidelines also highlight the importance of developing an individualized plan agreed upon by school and parents. This plan should ideally be amended annually. Throughout the set of guidelines, it is stressed that a collaborative approach is recommended and that successful diabetes management in school depends on effective communication with the family. It is, however, not made clear how to achieve and develop these.

The guidelines also state that all school personnel, including administrative staff, counsellors and nursing staff, should receive appropriate diabetes training [19].

In general, the guidelines focus primarily on what to do and not very specifically on how to do it. This is understandable to a certain extent, as the ‘how’ will depend on the specific contextual factors and structures in very different countries.

Based on the above combined findings from the initial phases of the KIDS study, and to a great extent on the contents and shortcomings of the ISPAD guidelines, the objective of the present study is to explore and develop possible solutions and recommendations for addressing the challenges and problems identified in the three needs assessment studies mentioned above.

## 2. Methods

### 2.1. Setting

The present study was conducted in Denmark, where the healthcare system operates across three political and administrative levels, namely the state, the regions and the municipalities. The five regions are primarily responsible for the hospitals, including the paediatric diabetes clinics and the 98 municipalities are responsible for a number of primary healthcare services [21]. Preschools and public elementary schools are under the administration of the municipalities. In Denmark, schools and preschools must provide the support required for children with T1D to attend school/preschool on an equal footing with other children [22]. Parents of children with T1D can apply to the municipality for additional non-trained support during school/preschool hours in the form of a special needs assistant (SNA) as well as for reimbursement for lost earnings and additional costs.

To make the best use of all the research findings and to ensure equal representation of all involved parties, we decided to conduct a comprehensive multistakeholder participatory workshop in each of the five Danish regions [23,24].

### 2.2. Participants and Recruitment

Relevant stakeholders were invited to take part in the workshop. We invited participants from all the people who had participated in the three needs assessment studies mentioned above. The three primary stakeholder groups represented were: 1. municipal administrative personnel, school and preschool personnel, 2. diabetes healthcare professionals (HCPs) from paediatric clinics and 3. families with a child with T1D. In the family group, we only included the children if they were older than 13 years of age. The distribution of stakeholders in each workshop is illustrated in Table 1.

### 2.3. Workshops

To ensure geographical spread, one workshop was conducted in each of the five regions in Denmark. All workshops were facilitated by the authors LBJ, AØN, MSI and DG, together with a local representative from the particular region in which the workshop took place.

Using workshops as a research method has the potential to create new insights and self-reflections among the participants through peer-to-peer discussions [23,24]. Each workshop was three hours in duration, took place at a local regional facility and concluded with all participants eating dinner together.

The workshops were designed to facilitate a reflective and mutual dialogue among the participating stakeholders. The dialogue was semi-structured and focused on how to provide assistance to children and young people with T1D in school and preschool settings, as well as to the parents and the school/preschool staff.

To facilitate the discussions, exercises took place with various group configurations. Initially, participants were divided based on the groups they represented. Subsequently, they were divided into mixed groups.

When composing the mixed groups the local facilitator focused intensely on creating diverse groups by considering diabetes knowledge, diabetes experience, gender, age, professional profile and overall work experience.

The workshop provided the participants with three exercises during which they were asked to:

- Share their own experiences related to support for children with diabetes, their families and/or the school/preschool personnel. This initial exercise gave the participants the opportunity to share success stories and problems with peers from the group they represented.
- Engage in discussions centred around real-life scenarios, examining the challenges encountered in everyday life, and the organization of diabetes support, from a different perspective than the one they represented. The scenarios were constructed based on the needs assessments.
- Propose concrete solutions to the problems discussed in the exercises and suggest improvements to the organizational support of children with T1D—all based on the knowledge shared during the workshop.

### 2.4. Analysis

The recorded workshop data were transcribed by the authors L.B.J. and A.Ø.N. The transcriptions summarized the group discussions and the participants’ points of view and included direct quotes to underpin the described data. First, the transcribed data were analysed manually by P.D., L.B.J. and A.Ø.N. and preliminary themes and subthemes were identified. Second, the data were transferred to the qualitative data analysis software Nvivo 14 for systematic analysis by P.D. and D.G. using content analysis and radical hermeneutics [25,26].

### 2.5. Ethics

At the workshops, written informed consent was obtained from all participants. According to The Danish National Ethical Committee, the present study did not require ethical approval. The study complies with the ethical guidelines laid out by the Declaration of Helsinki [27] and was approved by the Danish Regional Data Protection Agency (P-2021-276, 11-04-2021).

## 3. Findings

In total, 80 participants took part in the workshops, and all stakeholder groups were represented at each of the five workshops. Five main themes were identified in the analysis; the main themes and subthemes are illustrated in Table 2. In the following presentation of themes and subthemes, we have extensively limited the use of quotes from the workshops. The quotes that that we do present are therefore to be seen as examples.

### 3.1. Training and Knowledge

Diabetes training and knowledge were identified as major themes across the workshops. Participants found that schools and preschools lack knowledge and information when a child is returning to school after being diagnosed with T1D. The lack of knowledge was described as a barrier to providing an environment that feels safe for the child, the family and the staff members in schools caring for the child. Although all diabetes clinics in Denmark provide some form of diabetes training, the delivery of training varies across the country. In the workshops, it was discussed that not all clinics offered to train staff members at the child’s school/preschool, and that not all schools/preschools agreed to participate in the proposed training. Consequently, parents were often left with the responsibility and burden of facilitating the provision of needed diabetes knowledge to staff members. In this way, the quality of training depends on the parents’ resources and ability to facilitate and educate. Participants agreed that this approach was unreasonable and very fragile. The period following a diabetes diagnosis is typically very stressful for most parents as they are navigating uncharted territory with regard to diabetes management.

*“[…] As it is now, it’s the parents who are left with the responsibility for teaching and training. That’s really difficult—for all of us.”* (School staff member)

As this quote illustrates, the school staff are often aware that the parents are burdened by the responsibilities, but they see themselves as unable to take on some of the responsibilities due to lack of knowledge, training and support.

There was consensus among participants that professional training in diabetes care is needed for the successful inclusion of children with T1D in the school/preschool setting. It was proposed that HCPs from the clinics should establish a channel of communication directly with the school/preschool when a child is first diagnosed with T1D. It was recommended that the clinic should offer ongoing training, for example at a designated time each month, where staff members could visit or contact the clinic for continued training.

Across participants, there was agreement that the group of staff closest to the child should receive diabetes information and hands-on training from a specialized diabetes HCP. Further, to grasp the complexity of diabetes management and the responsibility involved, it was suggested that school management should participate in the training alongside staff members and parents. It was also suggested that the HCPs could use the setting to facilitate a future collaboration between the school/preschool and the home.

Participants agreed that apps, links and websites cannot replace the information and training that HCPs provide. In general, it was the parents’ experience that finding the needed information regarding diabetes organization and support is difficult. The participants requested standardized materials and information, provided by the diabetes clinic and aimed at parents, schools and preschools, respectively.

Participating teachers and care workers expressed their frustration concerning their lack of knowledge and competence regarding diabetes management. They explained that when staff members do not want to take on the responsibility for diabetes care, it is due to a feeling of insecurity. They pointed to a lack of quality in training and the constantly evolving technology as being the major challenges. Staff were worried about making mistakes and requested more hands-on, practical training. The importance of staff being trained in practical issues, such as how to replace a continuous glucose monitor that has fallen off, was highlighted.

Parents believed that appropriate training and knowledge would increase diabetes competences and decrease insecurities among staff members. Some suggested that staff members should receive training without the parents being present, to encourage an open discussion about difficult issues, such as feeling insecure in certain areas of diabetes management. Parents supported the idea of a forum for staff members to express insecurities about diabetes care, while having access to suitable educational support. It was proposed that diabetes HCPs would be able to provide this kind of ‘non-fear-driven’ training, whereas parents would be too emotionally involved.

“[…] If I then meet parents who are also insecure. We’re talking about a 3-year-old child who has had diabetes for a year. The parents, they look at me sometimes when I ask about a problem and then, you know…’well, I don’t know’. And I think, f**k! If you don’t know, how am I supposed to know?”—(Preschool care worker)

This quote sums up the vulnerable dynamics perfectly. When distressed and insecure parents are pressured into being diabetes experts, it is very likely that they will pass on their distress and insecurities to the preschool/school staff members, who will then be very reluctant to deal with the diabetes challenges.

Participants agreed that diabetes training should include knowledge about the social and emotional impact of diabetes, both in general and in relation to the individual child’s needs and preferences regarding support.

While diabetes technology is valuable, it was acknowledged that it also represents an increased psychological burden, as it encourages continual engagement with diabetes management. One mother experienced the misconception that technology ‘takes care’ of diabetes and that it negates the need for support. Constantly being able to track blood glucose and getting reminders were experienced as difficult.

One young person described it as difficult when other people do not know about diabetes and the technology.

*“When it beeps, they often go: ‘Seriously, can you put that on silent!?’. ‘no, I can’t do that’.”*—(Young person with diabetes)

Children and young people with T1D should feel safe at school, knowing that an adult is around to help with diabetes-related challenges. It was discussed how some children and young people might want more support but do not express this in the situation. Some children do not wish to be a burden, disturb activities during class, or find it difficult to express when they feel unsafe. Therefore, it was suggested that an individualized plan should be created, based on the needs and preferences of the child.

### 3.2. Communication and Collaboration

Good communication and a well-functioning partnership between the home and school/preschool are paramount for diabetes management and the overall wellbeing of the child during school/preschool hours. Parents explained how a lack of communication creates a feeling of insecurity, and teachers and care workers explained that they feel unsafe if clear communication from their management is lacking. Participants identified that maintaining an ongoing adaptive conversation, engaging in shared decision-making and having realistic expectations were central components. Additionally, recognition of the scope and responsibility of managing diabetes was important to facilitate a strong partnership.

The importance of honest, respectful communication was emphasized in the situations where technology is used to share data between the home and the school/preschool. This concerned, for instance, staff members having access to the child’s blood glucose readings outside the hours that they care for the child and parents tracking their child’s blood glucose data via their phone. Thus, addressing or referring to these data requires trust and must be approached in a sensitive and respectful way to avoid a breakdown of communication or even the discontinuation of data sharing.

*“But surely that’s also why collaboration, or partnership with the parents, is extremely important? If that is in place, you can do much, much more. Both as parents and as staff. And this applies to diabetes and everything else too”* (parent)

As this quote illustrates, both the parents and the staff agreed that collaboration was the way to successfully deal with challenges and frustrations. However, they did not know how to facilitate this collaboration as the roles and responsibilities were unclear to both parties.

One suggestion was the importance of arranging an initial network meeting shortly after diagnosis. This meeting would serve to facilitate communication, establish common expectations and clarify responsibilities. It was suggested that the family, HCPs, school/preschool and relevant personnel from the municipality should attend such meetings. Network meetings were believed to be particularly useful at diabetes onset and during transitions. Some parents felt that the communication fizzled out quickly, and they advocated for an ongoing dialogue beyond the initial period surrounding the diagnosis. Many agreed that follow-up meetings are important. The child’s requirements change, and it takes some time to understand how diabetes impacts the child and the family. Participants talked about the possibility of relevant people meeting a few times a year to share knowledge in a format similar to the present workshop.

As a tool to facilitate the clear communication of expectations, the participants agreed that an action plan should be created when a child is diagnosed. The plan should be agreed upon by the child’s parents, the school management and key staff members. Incorporating school management into the planning process was considered vital, as they have the overarching responsibility but might not possess a complete understanding of the complexity and extent of diabetes management.

Depending on the child’s age, it is important to include the young patient with diabetes in the planning. This inclusion is crucial to ascertaining the type of support required and identifying what the child/young person finds beneficial. It was suggested that having a clear plan may prevent situations where the parents cannot send their child to preschool/school due to a lack of resources or competent staff members.

The participants discussed the importance of establishing clear responsibilities in the context of diabetes management. There was consensus that the schools and preschools should take on the responsibility for providing support for diabetes care during school hours. However, participants mentioned a lack of knowledge and a feeling of insecurity among staff members as barriers to fully assuming this responsibility. Hence, the participants emphasized the significance of having a well-defined division of responsibilities, clarity about these responsibilities and the appropriate training. Roles and responsibilities may change over time, in line with the child’s development and changing circumstances, which necessitates ongoing communication.

*“If no one will take on the responsibility at school, then you can make all the websites you like! It will never work”* (Father)

The fact that most parents will invariably have concerns about their child and will remain readily available by phone throughout the day was discussed. This has been reinforced by the latest technology. However, clear divisions of responsibility should prevent unnecessary stressful situations for parents, children and staff members. Without the clear allocation of responsibility, there is a risk that children will be left without the needed support to effectively manage their diabetes while at school. Because of the current lack of support during school hours, many parents explained that they must be available by phone to help their child throughout the day. This was described as a significant stress factor for many parents.

The discussion revealed that parents are frequently uncertain about who to contact and the specific roles and responsibilities of different individuals. Staff members are uncertain about their own roles and responsibilities, and they may be unclear about when they can decline responsibility for a child, especially when they are in doubt and unsure about whether their actions are correct. Consequently, the participants requested the development of national guidelines that comprehensively outline and delineate the roles and responsibilities of diabetes care.

### 3.3. Designated Contact/Support Person

A lack of resources and too few staff members trained in diabetes management were highlighted as the main factors hindering schools and preschools from caring for children with diabetes during the early or late hours of the school day, or in situations when trained staff members were unavailable.

Participants agreed that having a designated person in school who is responsible for coordinating the child’s care during school hours would be beneficial. Although the entire group of staff caring for the child should all have received training in handling diabetes, one person should be designated as the primary contact/support person. Participants commented that the contact person should have more diabetes knowledge and be responsible for continually educating relevant colleagues. In this way, parents would not have to teach repeatedly when staff members were sick, on leave, or otherwise unavailable. The contact person would also be available to answer questions when other staff members require help with diabetes-related issues. Several participants suggested the need for a primary and secondary contact person.

The exact role of the contact person should be clearly defined. The participants suggested that the contact person should facilitate communication between the parents and the school and ensure that important information from the home always reached relevant staff members. For younger children, the contact person could track their blood glucose data via an app. For older children, it was suggested that the contact person should still have adequate diabetes knowledge, so that the child feels confident about getting help with managing blood glucose and go about turning to the contact person when in doubt about diabetes.

*“So even in the 6th grade I still had a contact person that I went down to every time we had food. Her name was […] and she was amazing”*—(Young person with T1D)

In this way, for older children, the contact person would not necessarily be physically present in the classroom, but available and able to help with regulating blood glucose. It was suggested that the contact person would be responsible for practical aspects necessary for the child’s safety, e.g., making sure snacks and equipment are packed for school outings and being the responsible adult on overnight school trips.

It was proposed that a designated contact person could gradually build a trusting relationship with the child and provide psychosocial support, based on the individual child’s needs and preferences. This was believed to improve diabetes management during school hours, as it was evident from the discussion that some children were not comfortable sharing their diabetes-related challenges with their teachers. Some children had experienced not receiving any useful support. As one young person shared with the group:

*“I would like the teachers to maybe just get an alarm, maybe just ask if I was okay. Because when I get low, I don’t really say that I get low or anything. Then I might feel really bad. But if you pull yourself together and tell your teacher, they would sit you with your friends... and well, my friends didn’t really know what to do either.”*—(Young person with T1D)

This quote sums up the importance of having someone relatively close to the child who has received basic training and who is able to provide psychosocial support and thereby also ease the stress and insecurities of the individual teachers.

### 3.4. National Guidelines

Participants agreed that there is a need for national alignment to minimize the differences in diabetes support.

Applying for support, in the form of special needs assistance (SNA) and/or reimbursement for a loss of earnings, requires resources from parents as well as administrative resources from the school/preschool. Parents explained that they must continually apply (twice yearly) for SNA for their child. They described the process as stressful and non-transparent. The participants expressed their frustration over the many ‘boxes’ in which families with diabetes can be placed in the municipality, and parents were generally unsure of their rights, regarding what they are entitled to. For many this creates confusion and a sense of being pawns in a non-transparent system. The participants believed that personal resources, parental persistence and an understanding of the law were perceived to influence the family’s ability to secure the needed support for their child.

In the workshops, participants discussed how the lack of support affects families differently. Some parents have a flexible work environment and can provide support for their child during school hours, others cannot. For some parents, the situation threatens their ability to work and ultimately results in the inequality of treatment for children with T1D. Likewise, diabetes training offered to schools/preschools differs across regions and municipalities. For example, regional differences determine whether HCPs from the child’s diabetes clinic will visit the school/preschool, whether classmates are offered diabetes training and whether re-training is offered at transitions, etc.

Participants requested a fair and more transparent system, where diagnosis with T1D would give rise to a structured course of action. For this to materialize, many participants called for the creation of national guidelines. In general, the participants suggested that national guidelines would simplify the process of securing adequate support for children and assist families and schools/preschools in navigating the system.

It was proposed that national guidelines should contain a clear allocation of responsibility, i.e., what responsibilities fall upon the parents, the schools/preschool, the diabetes clinic and the municipality.

Participants explained that the complexity of diabetes management is not recognized and that it often is not made clear how great a task managing diabetes is going to be for the school/preschool. While parents experience goodwill on the part of staff members, as regards taking on diabetes management tasks, a lack of time and resources sometimes prevent staff from adequately supporting children with diabetes, as schools often have to finance the need for more staff themselves. Some parents had experienced situations in which very young children were expected to handle diabetes independently.

Participants agreed that extra resources, in the form of SNA, are needed for schools/preschools to care for a child with T1D and that the need is greater for young children in preschool. It was suggested that such support should be granted universally at diagnosis and be based on the child’s age. In addition to the standard allocation of support, an individual assessment of the child and circumstances could be carried out. The consensus among participants was that children under 12 years of age need extra support during school hours and that this should be supported financially.

*“The fact that you have a child who needs something extra, and you can see that the other children... the fact that your child’s needs take a huge amount of resources from the other children, because there’s no extra support. So, I think, damn it, that makes me feel guilty as a parent!”*—(Parent)

This quote highlights the other significant challenge for institutional environments where the staff have to use significant resources to take care of a child with T1D without receiving any kind of added support. The other children will inevitably be neglected, which will then generate even more frustration and feelings of guilt among parents and staff members. National standards for providing added support for the school/preschool would prevent this situation from occurring.

### 3.5. Diabetes Coordinator

Currently diabetes-related issues are placed under various administrations in the municipality. Participants described how the process of navigating the different administrations within the municipality requires a great number of resources, leaving some parents with an experience of being tossed around the system.

For this reason, many participants called for a diabetes-specialized team in the municipality or a ‘diabetes coordinator’ who can be contacted for guidance on all aspects of diabetes-related support. The coordinator would have knowledge and access to relevant information regarding diabetes organization and support and would provide stakeholders with a single entry to the municipality.

It was suggested that the coordinator should be notified when a child is diagnosed. They would have an overview of what support is available for the child and the family and have the necessary knowledge to create transparency for parents navigating the process. This would include assistance with applications for SNA and/or reimbursement of a loss of earnings.

It was suggested that there should be a taskforce across teams in the municipalities, similar to what already exists for children with dyslexia and children born prematurely. Some proposed that a municipal health visitor could have this function. The role could include responsibility for the coordination between the diabetes clinic, school/preschool and the parents, and the health visitor would attend and coordinate network meetings.

## 4. Discussion

This ideation part of the Danish KIDS study set out to generate ideas for how to address the specific challenges identified in the initial needs assessment studies. Most importantly, we wanted to address the challenges associated with a lack of structured communication and the ensuing confusion and misunderstandings regarding roles and responsibilities [8,9,18]. Apart from these needs assessment results, it was also important to remain in line with—and thereby expand directly upon—the existing ISPAD guidelines [19,20].

Our results from the present ideation study emphasized with even more clarity that communicative structures and dynamics are at the very core of the identified problems and challenges, and that the possible solutions therefore should also revolve around improving existing structures and highlighting the importance of constantly working on understanding and developing communication strategies.

The decision to include all the involved parties in the same workshops was a calculated risk. We were initially unsure about whether the groups of workshop participants would be too large or too diverse. Presumably because of thorough planning and the participatory and inclusive nature of the workshops, they worked out extremely well and gave us a rich and detailed dataset.

While all the results and themes outlined above are important for a full understanding of the needs and possible ways to address these needs, using the results to develop a concise set of implementable recommendations for practice was an important step. This means that even though all the results mentioned above played a significant role in creating a full understanding of the potential aspects to be addressed, only relatively few of them are explicitly mentioned in our proposed set of practice recommendations outlined below.

## 5. Recommendations for Practice

Based on the initial needs assessment and the results from the ideation phase outlined above, we have developed a set of concrete recommendations to improve the communicative process that would ideally be initiated at the time of diabetes onset:

The main overarching recommendation is to write a concrete action plan at the time of diagnosis. The main objective of this plan is to align expectations regarding communication, training and support, and determine how responsibilities should be shared among stakeholders, i.e., the child, parents, school/preschool staff, HCPs and municipal administrative staff. This plan should be the main topic of a network meeting that is to be scheduled with all these parties within the first 2–3 weeks after diagnosis.

To meet this recommendation, we suggest the following steps and elements:

- At the time of diagnosis, the diabetes clinic informs the municipality. This information should go directly to one designated person or unit within the municipal administration who knows about diabetes and the process that will begin.
- Every municipality has a distinct and accessible organizational placement of the employees who are responsible for helping families and/or school and preschool staff in diabetes matters.
- The diabetes clinic is responsible for organizing the network meeting with the child (depending on age), parents, school/preschool staff, HCPs and municipal administrative staff.
- At the network meeting, the parties fill in (and sign) a cooperation agreement.
- A diabetes contact team is established at the school/preschool. This team is responsible for everyday diabetes management support. A member of the team should be the person with primary responsibility.
- All clinics/hospitals are responsible for training the school/preschool staff who are in daily contact with the child with diabetes, preferably on the school/preschool premises.
- Training should also be carried out if the child changes schools and at important transitions during institutional/school years.

## 6. Conclusions

The KIDS recommendations are primarily to be regarded as a supplement to the existing ISPAD guidelines. There is only one significant point on which the recommendations differ slightly: the current ISPAD guidelines state that it is the parents’ responsibility to communicate the diagnosis to the school personnel. In the recommendations above, we state that the communication process entails that it is the diabetes clinic that informs the municipality at the time of diagnosis and that this initial notification then starts the process of communication, which is characterized by clear roles and understandable responsibilities. This will remove some of the initial responsibilities from the parents and hopefully eliminate many of the cases of misunderstandings and communicative dead ends.

The next step will potentially (and depending on further funding) be to implement the recommendations across the five Danish regions and to evaluate the effects. The primary outcome will be parental distress, measured by the questionnaire Problem Areas In Diabetes for parents (P-PAID-C) [28]. Other measures will include the family impact survey, absence from work, and measures of parental socioeconomic backgrounds in order to examine the possible differential impact. The main secondary outcome will be the proportion of time spent in the target glycaemic range among the children, especially during time spent at school.

Even though our recommendations are specific to the Danish context, they are developed based on existing ISPAD guidelines and are therefore somewhat transferable to countries with similar healthcare setups. To make them transferable to other countries it will take local assessment processes to ensure that the problem areas and overall communicative barriers and potentials are comparable. We do, however, believe that the recommendations will be beneficial and implementable in places with similar challenges.

## Figures and Tables

**Table 1:** Participant distribution in the 5 regional workshops.

	1. North	2. Central	3. South	4. Zealand	5. Capital	Total
Municipal, school, and preschool personnel	4	10	5	9	7	35
Paediatric diabetes clinicians	5	3	4	3	2	17
Families with a child with T1D (parents + young people)	6 + 3	2 + 0	5 + 2	4 + 3	3 + 0	28
Total	18	15	16	19	12	80

**Table 2:** Themes and subthemes of the analysis.

THEMES	SUBTHEMES
**1. TRAINING AND KNOWLEDGE**	Diabetes training from specialized HCPs Re-training Diabetes competency and technology Lack of knowledge leads to insecurities Psychosocial aspects of diabetes
**2. COMMUNICATION AND COLLABORATION**	Network meetings Technology and data sharing Responsive communication, including the child’s perspective Action plans Roles and responsibilities
**3. DESIGNATED CONTACT/SUPPORT PERSON**	Relationship and trust—parents and child Sharing diabetes knowledge Primary contact for parents Diabetes management support and psychosocial support
**4. NATIONAL GUIDELINES**	Transparency of diabetes support Fair and transparent system Support in school for all children Financial support to preschool and school
**5. DIABETES COORDINATOR**	A key person at the municipal level Coordinator across municipal teams A single entry point to the municipality Knowledge on all aspects of diabetes-related support Coordination between clinic, school/preschool and parents

## Data Availability

Data are unavailable due to the language barrier and the need for anonymity.
